# Supplementary figures and images for: Label-free deep learning-based species classification of bacteria imaged by phase-contrast microscopy
Source: PLoS Comput Biol. 2023 Nov 13;19(11):e1011181. doi: 10.1371/journal.pcbi.1011181 (PMC10681317; doi:10.1371/journal.pcbi.1011181)

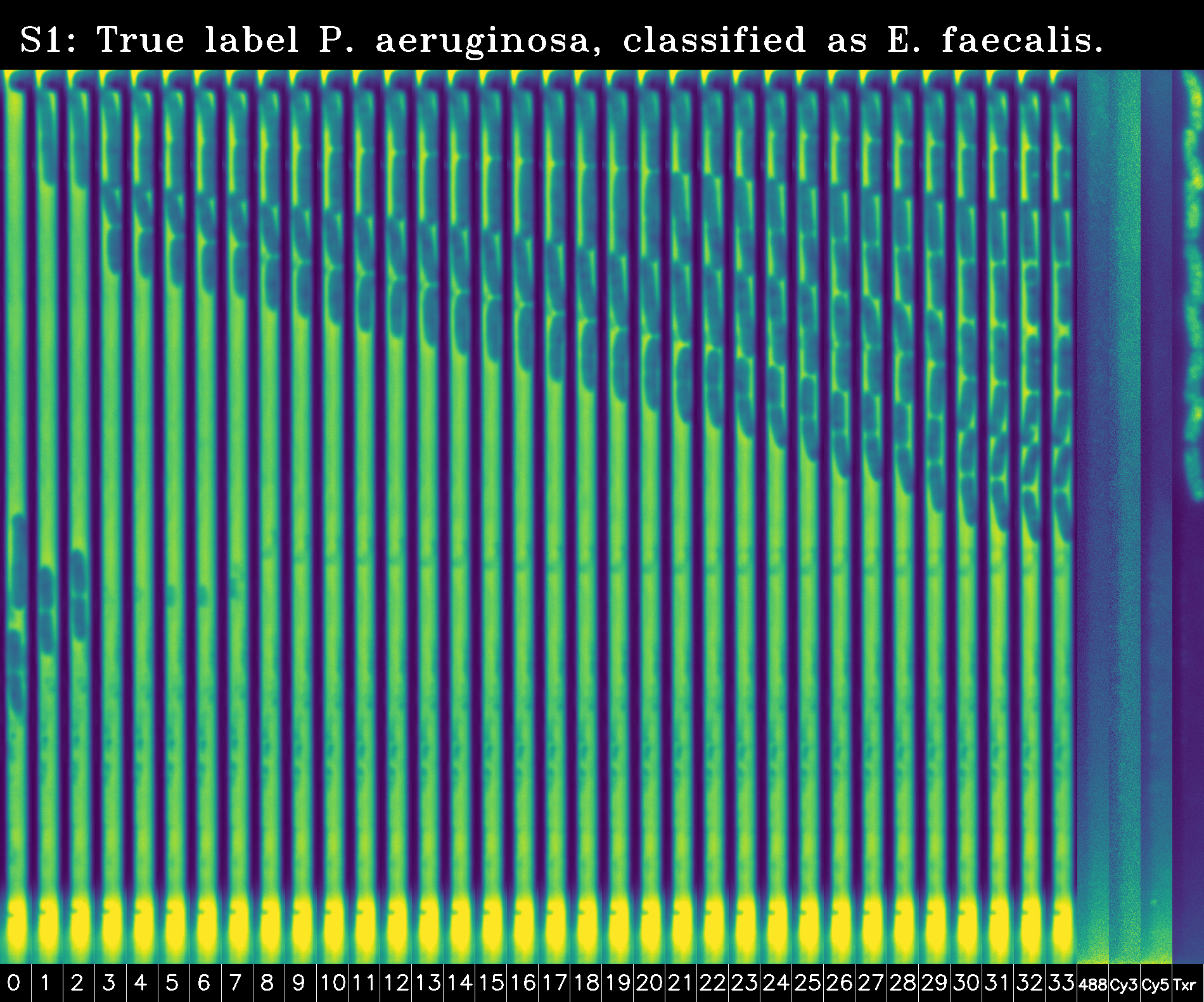

Supplement: S1 Fig — True label P. aeruginosa, classified as E. faecalis. The ResNet may have confused the stop at the top of the trap as a coccus. The upper half of the trap was empty in the first frame. (TIF) [file pcbi.1011181.s001.tif]

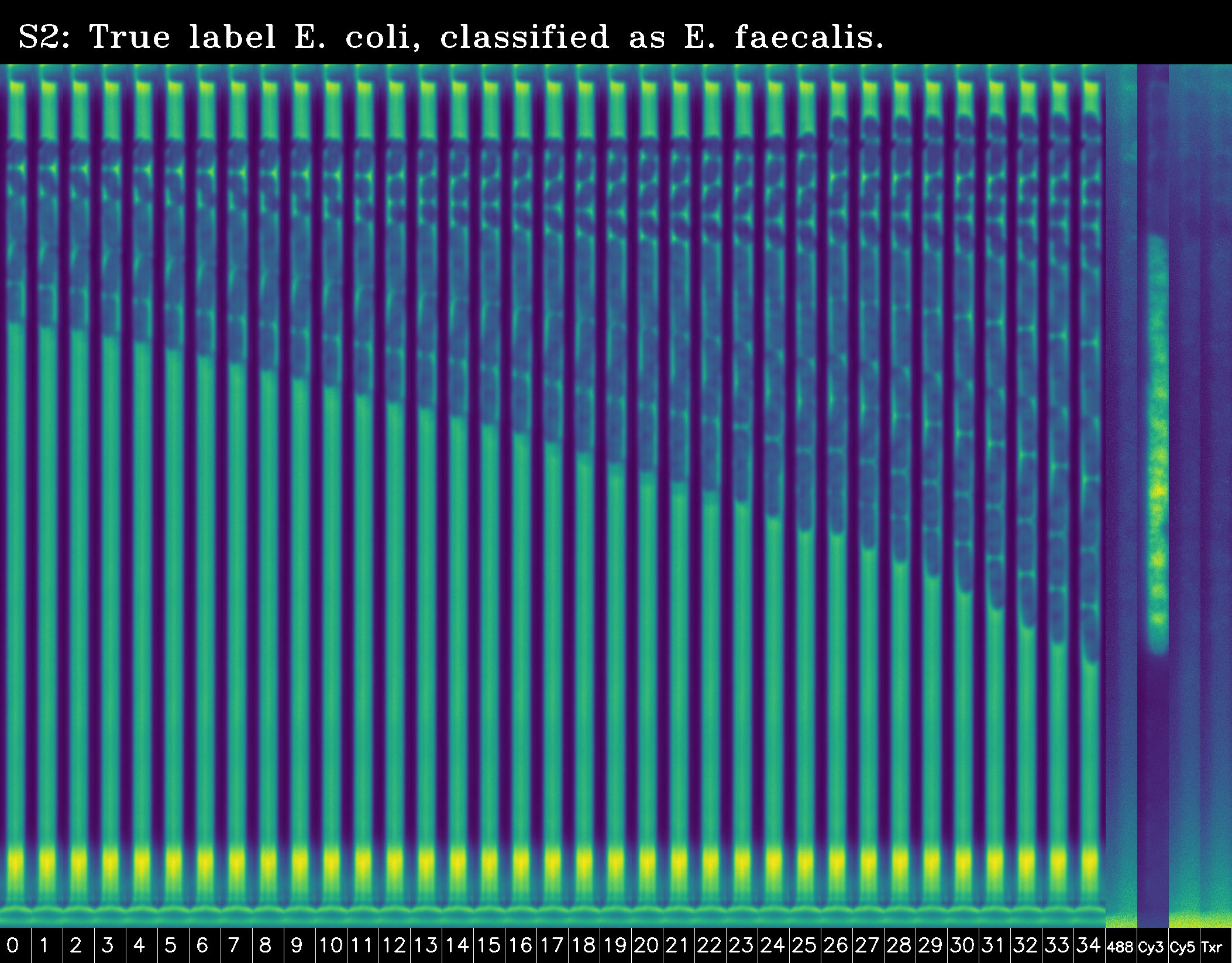

Supplement: S2 Fig — True label E. coli, classified as E. faecalis. It appears to have been cocci in the trap that avoided staining, and the trap clearly did not only contain a single bacterial species. (TIF) [file pcbi.1011181.s002.tif]

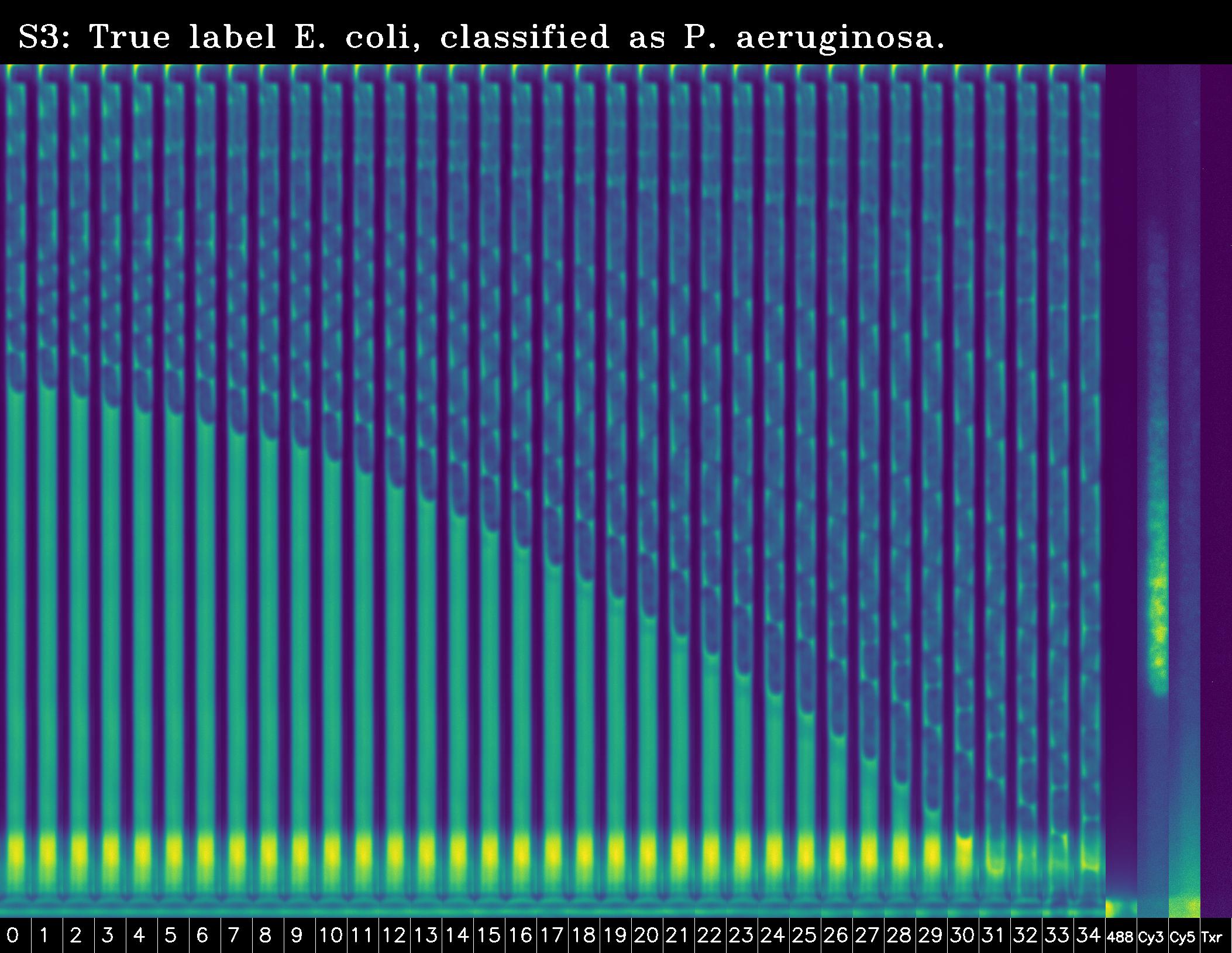

Supplement: S3 Fig — E. coli, classified as P. aeruginosa. Both species are rods with similar shapes and are easily confused, and the fluorescent staining indicates that there may be two bacterial species in the trap, where one has avoided staining. (TIF) [file pcbi.1011181.s003.tif]

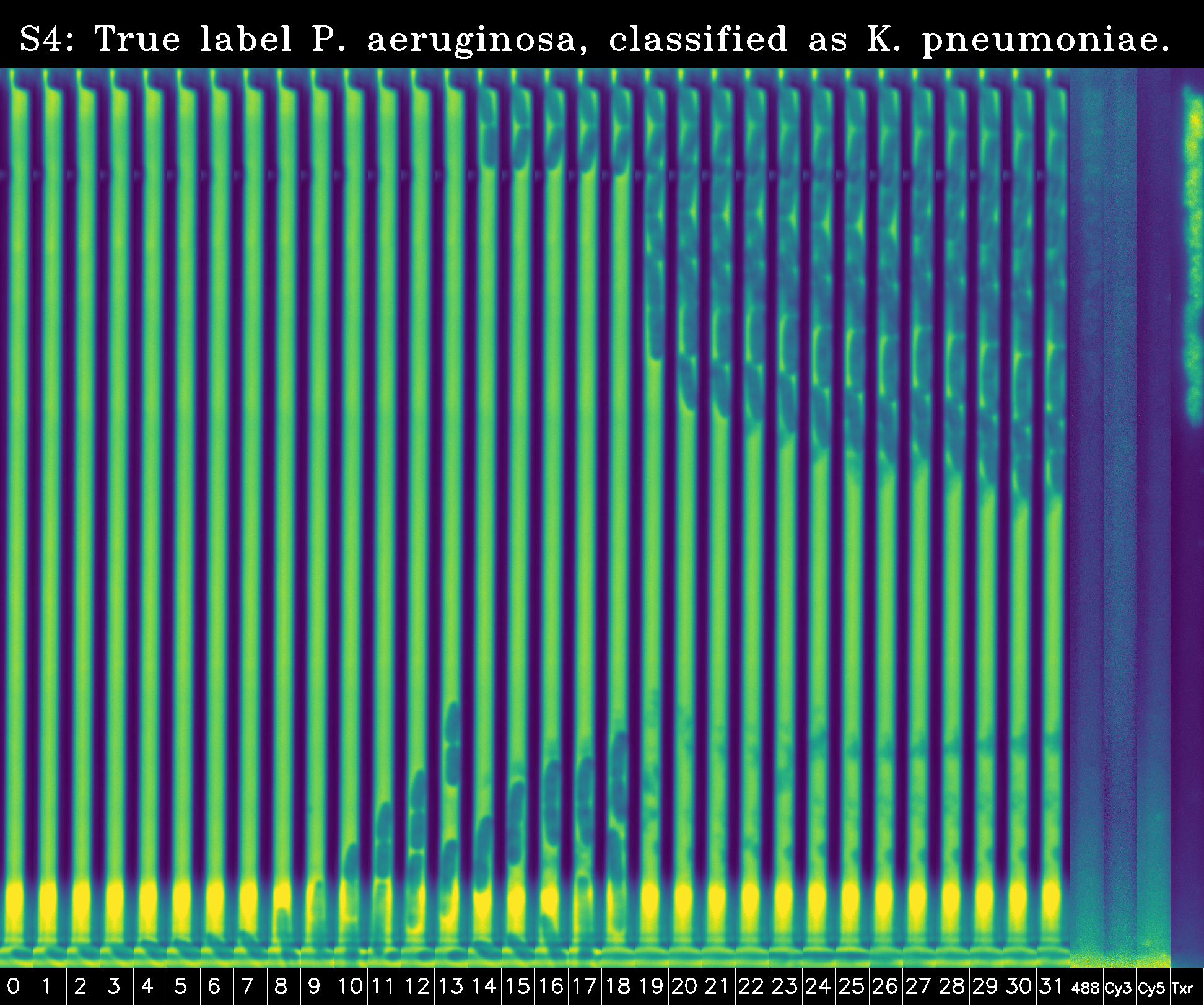

Supplement: S4 Fig — True label P. aeruginosa, classified as K. pneumoniae. The trap was empty in the first frames. (TIF) [file pcbi.1011181.s004.tif]

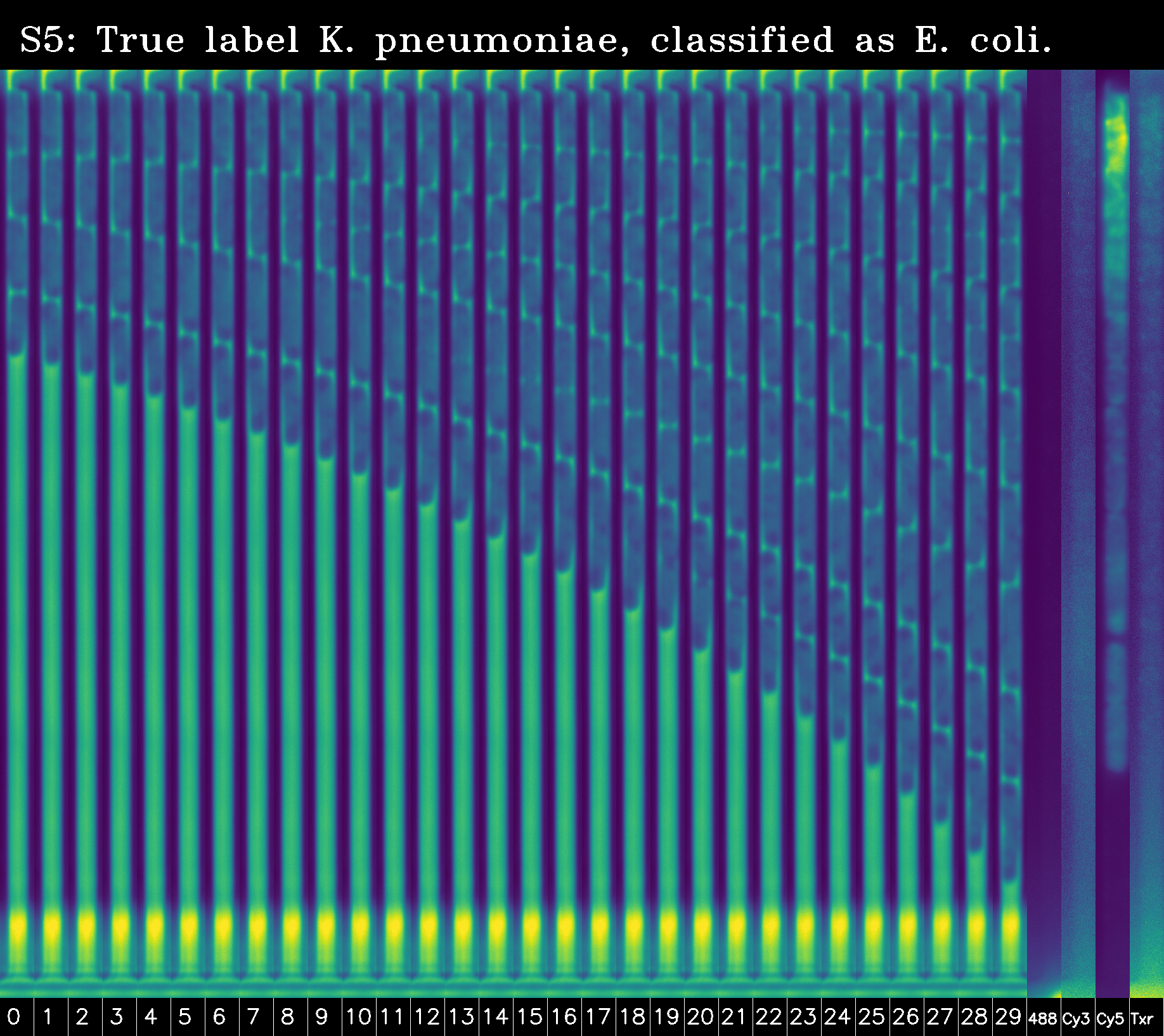

Supplement: S5 Fig — True label K. pneumoniae, classified as E. coli. Both species are rods with similar shapes and are easily confused. (TIF) [file pcbi.1011181.s005.tif]

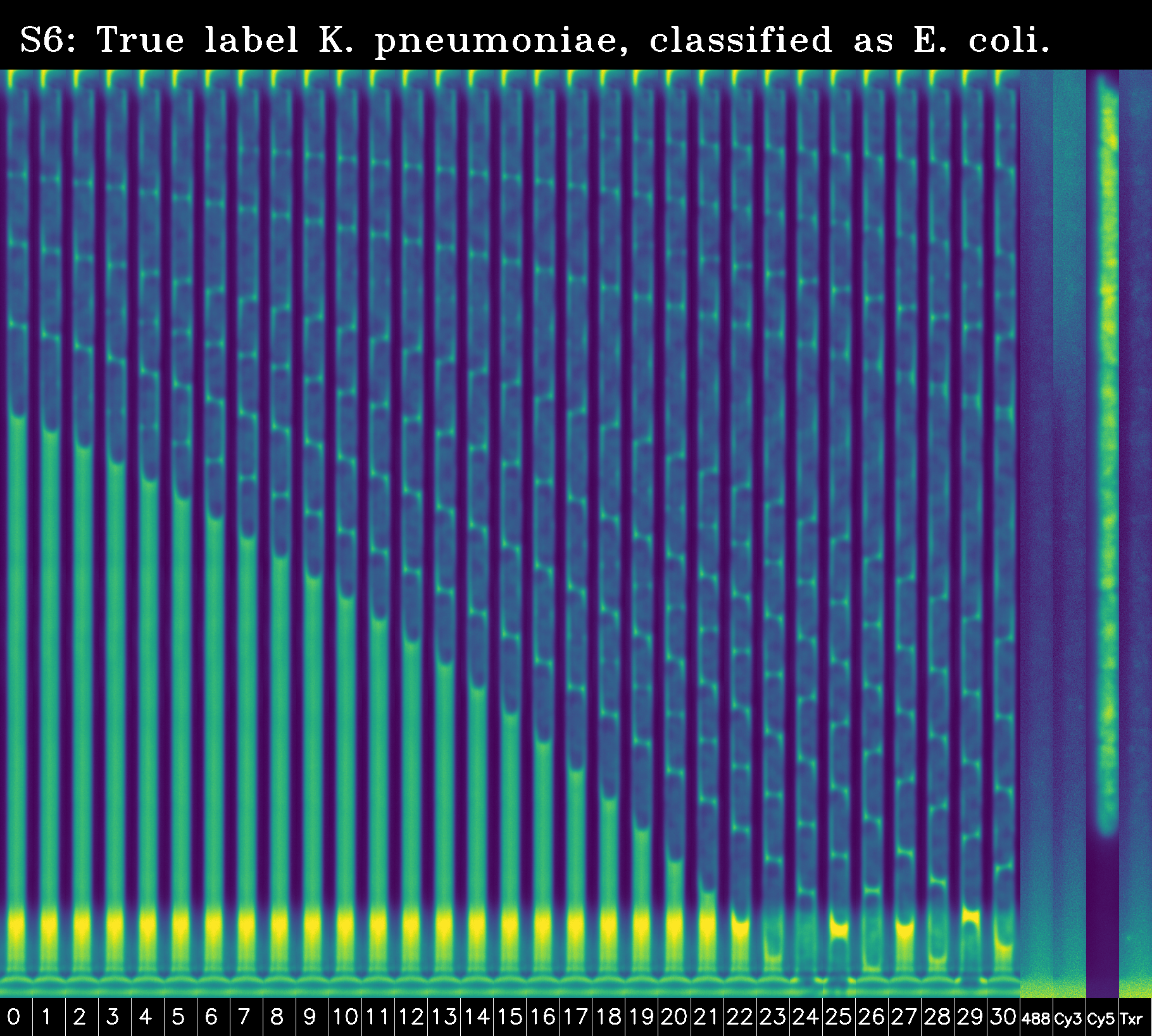

Supplement: S6 Fig — True label K. pneumoniae, classified as E. coli. Both species are rods with similar shapes and are easily confused. (TIF) [file pcbi.1011181.s006.tif]

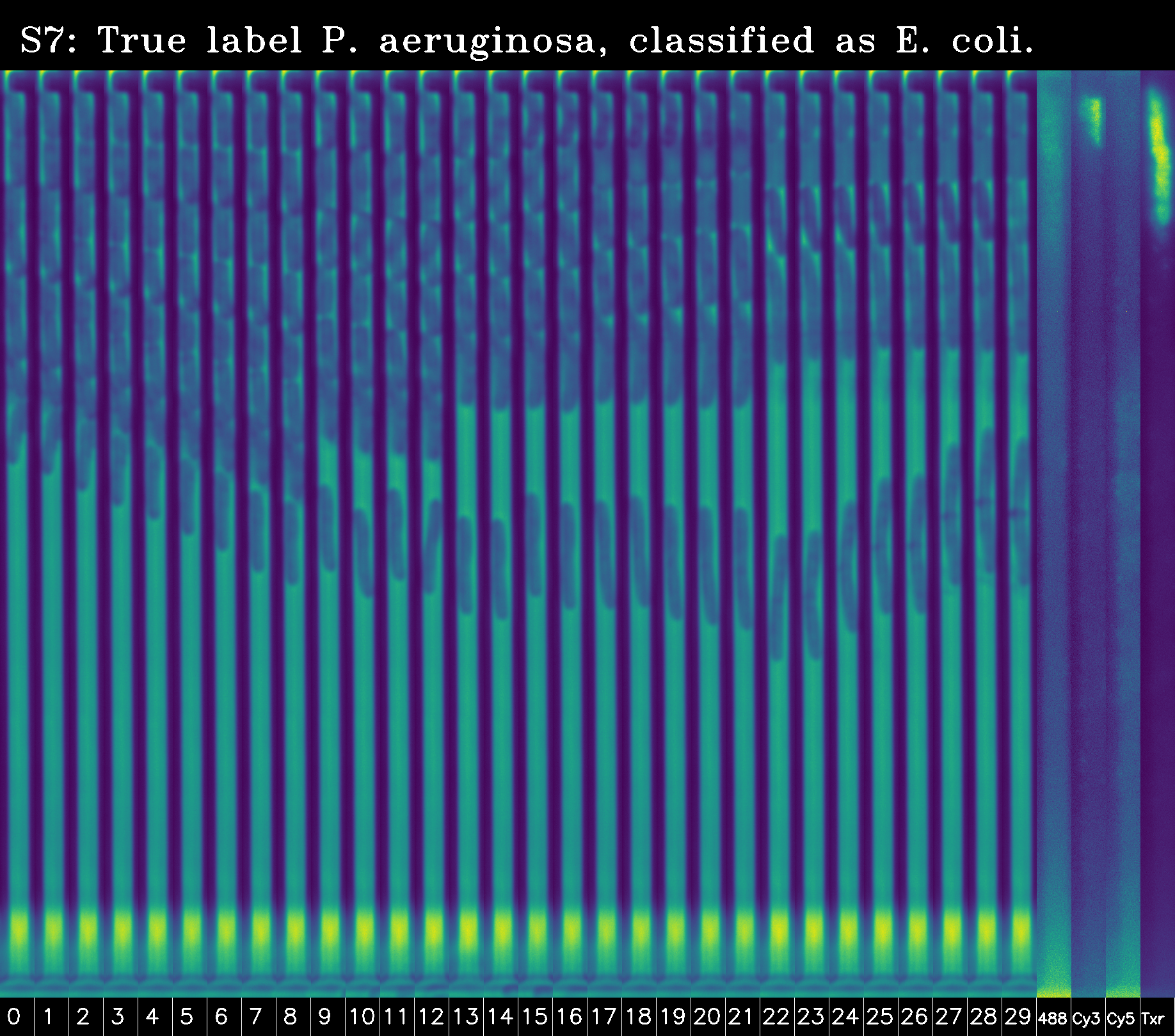

Supplement: S7 Fig — True label P. aeruginosa, classified as E. coli. Both species are rods with similar shapes and are easily confused. It appears to have been several species in the trap that avoided staining under heavy antibiotic treatment. (TIF) [file pcbi.1011181.s007.tif]

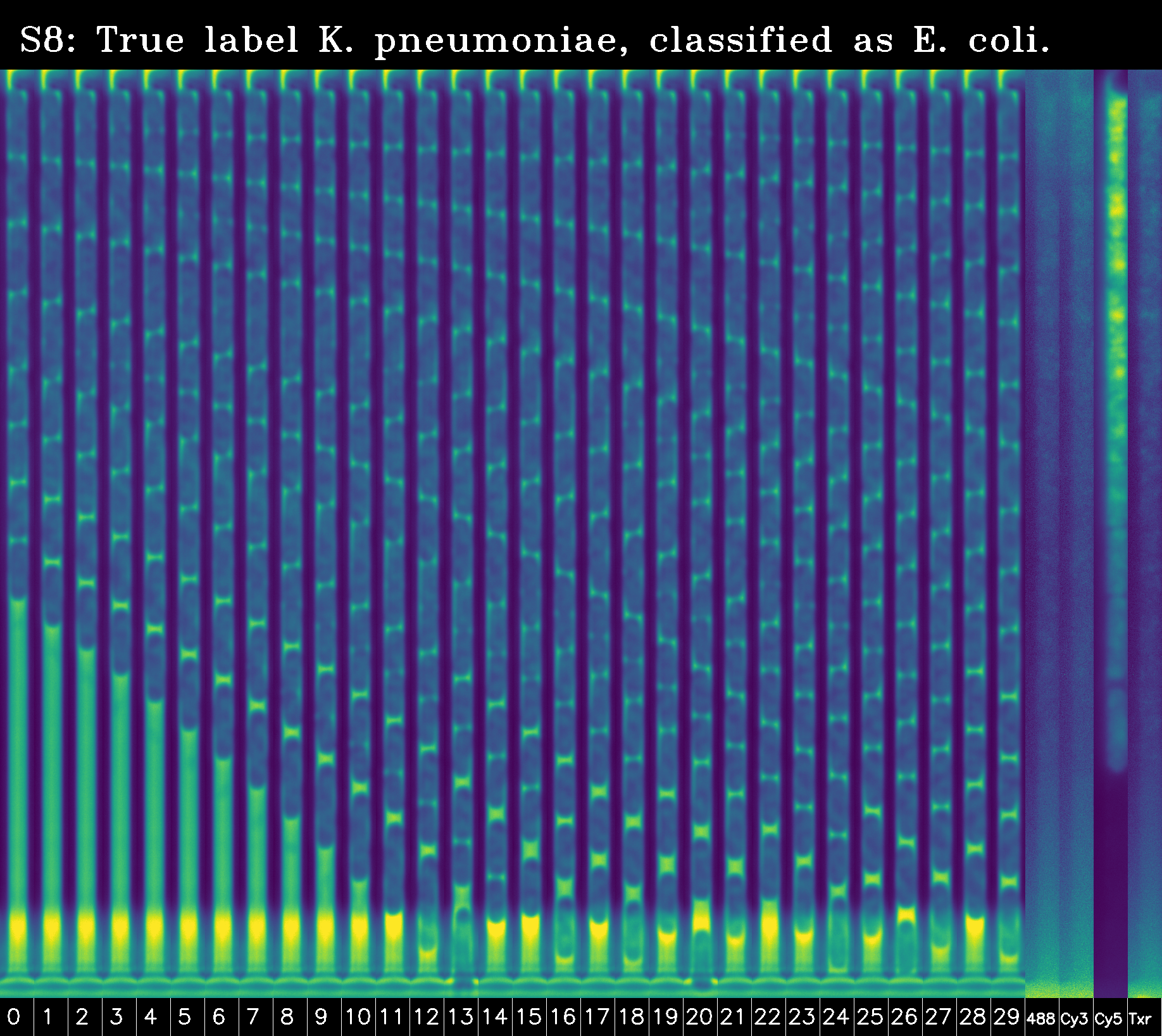

Supplement: S8 Fig — True label K. pneumoniae, classified as E. coli. Both species are rods with similar shapes and are easily confused. (TIF) [file pcbi.1011181.s008.tif]

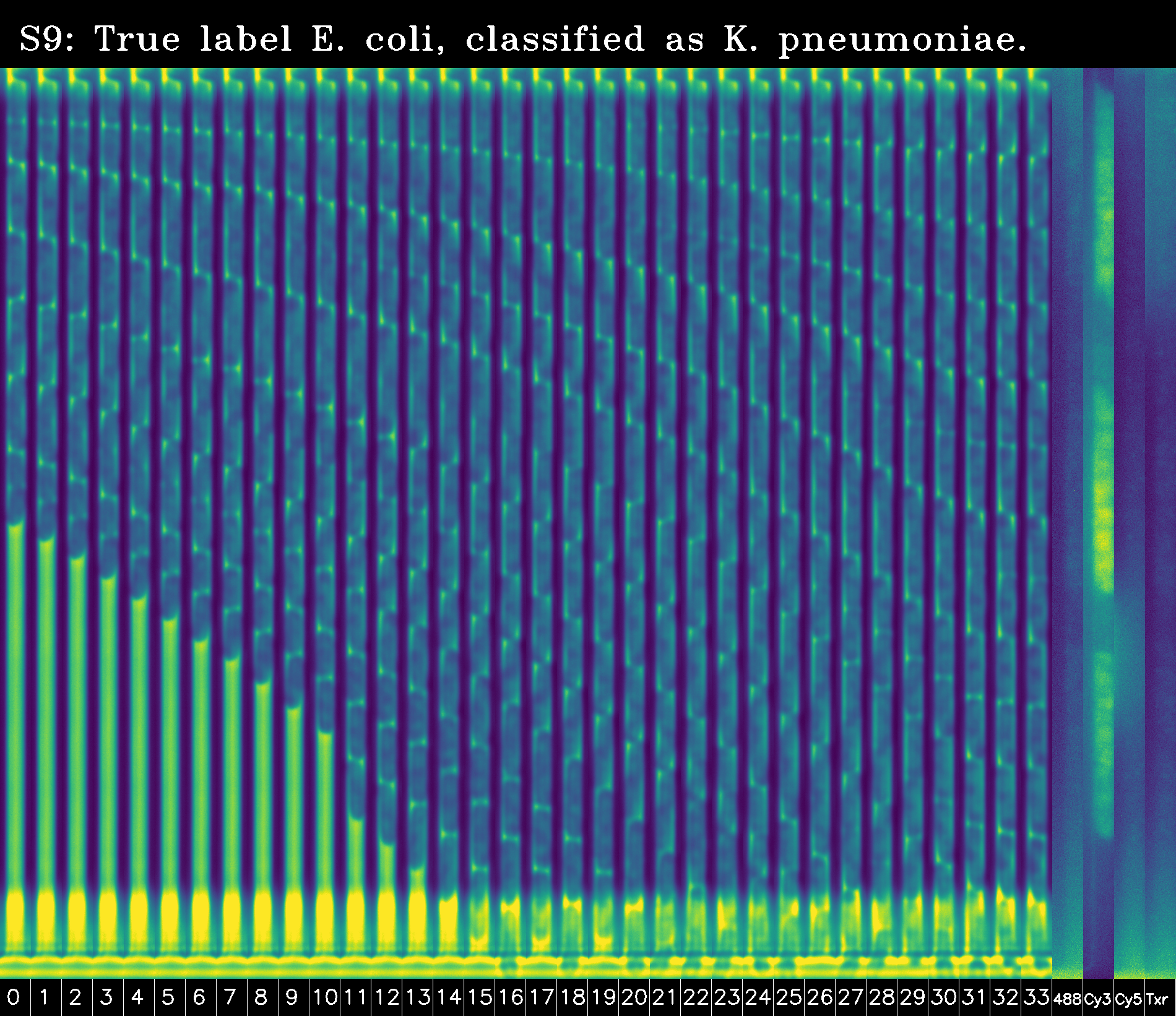

Supplement: S9 Fig — True label E. coli, classified as K. pneumoniae. Both species are rods with similar shapes and are easily confused. (TIF) [file pcbi.1011181.s009.tif]

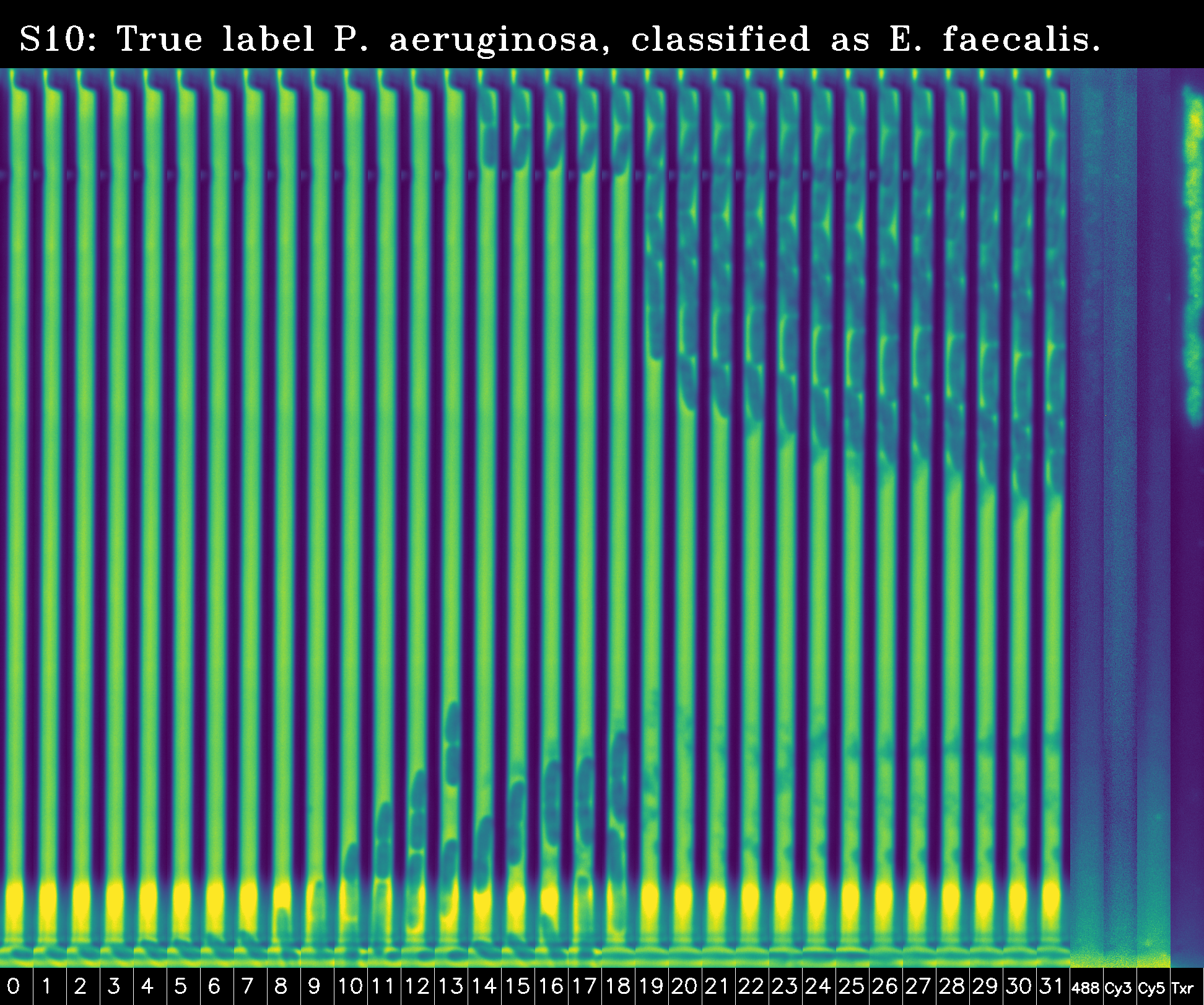

Supplement: S10 Fig — True label P. aeruginosa, classified as E. faecalis. The ViT possibly confused the stop at the top of the trap as a coccus. The trap was empty in the first frame. (TIF) [file pcbi.1011181.s010.tif]

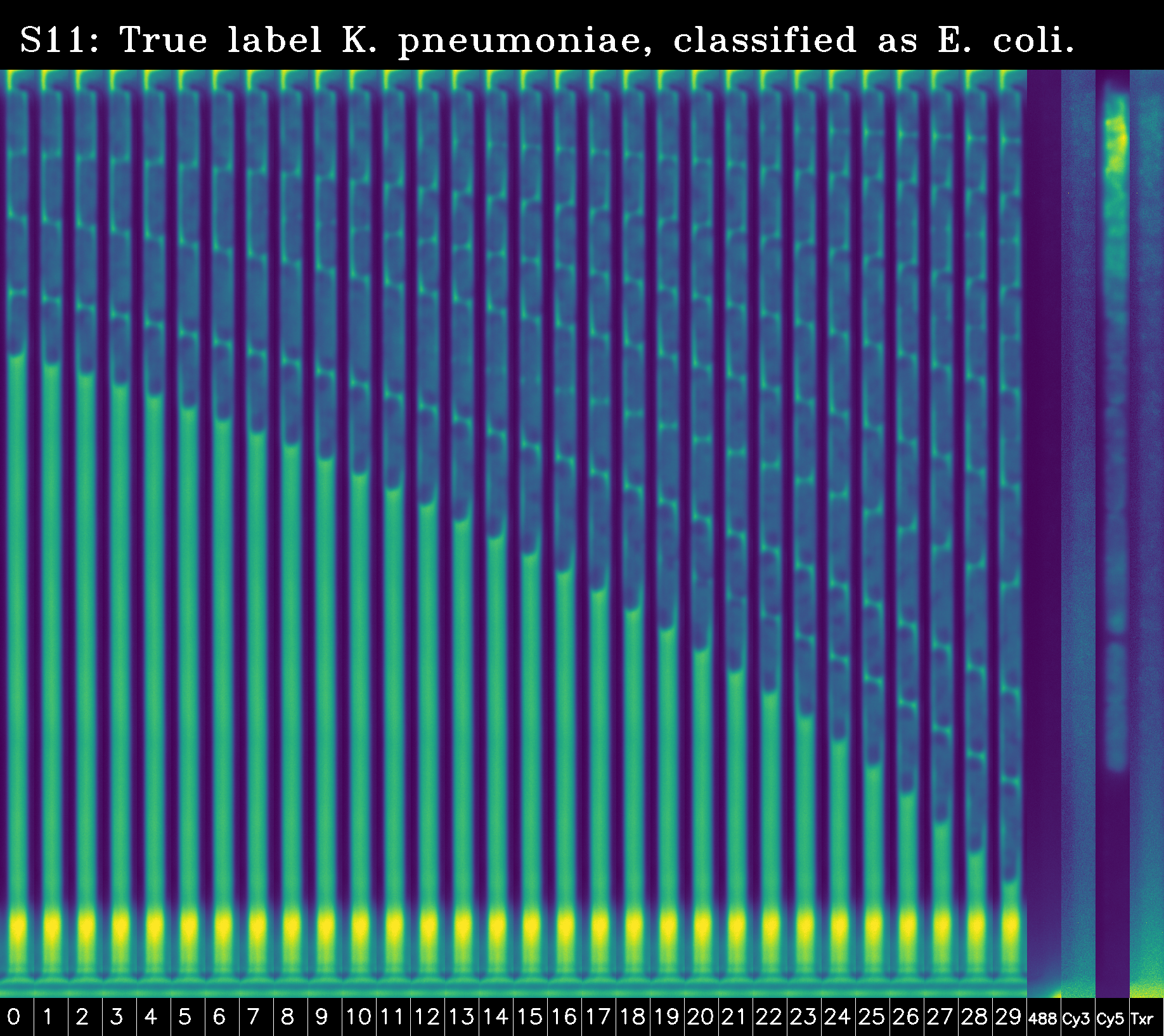

Supplement: S11 Fig — True label K. pneumoniae, classified as E. coli. Both species are rods with similar shapes and are easily confused. (TIF) [file pcbi.1011181.s011.tif]

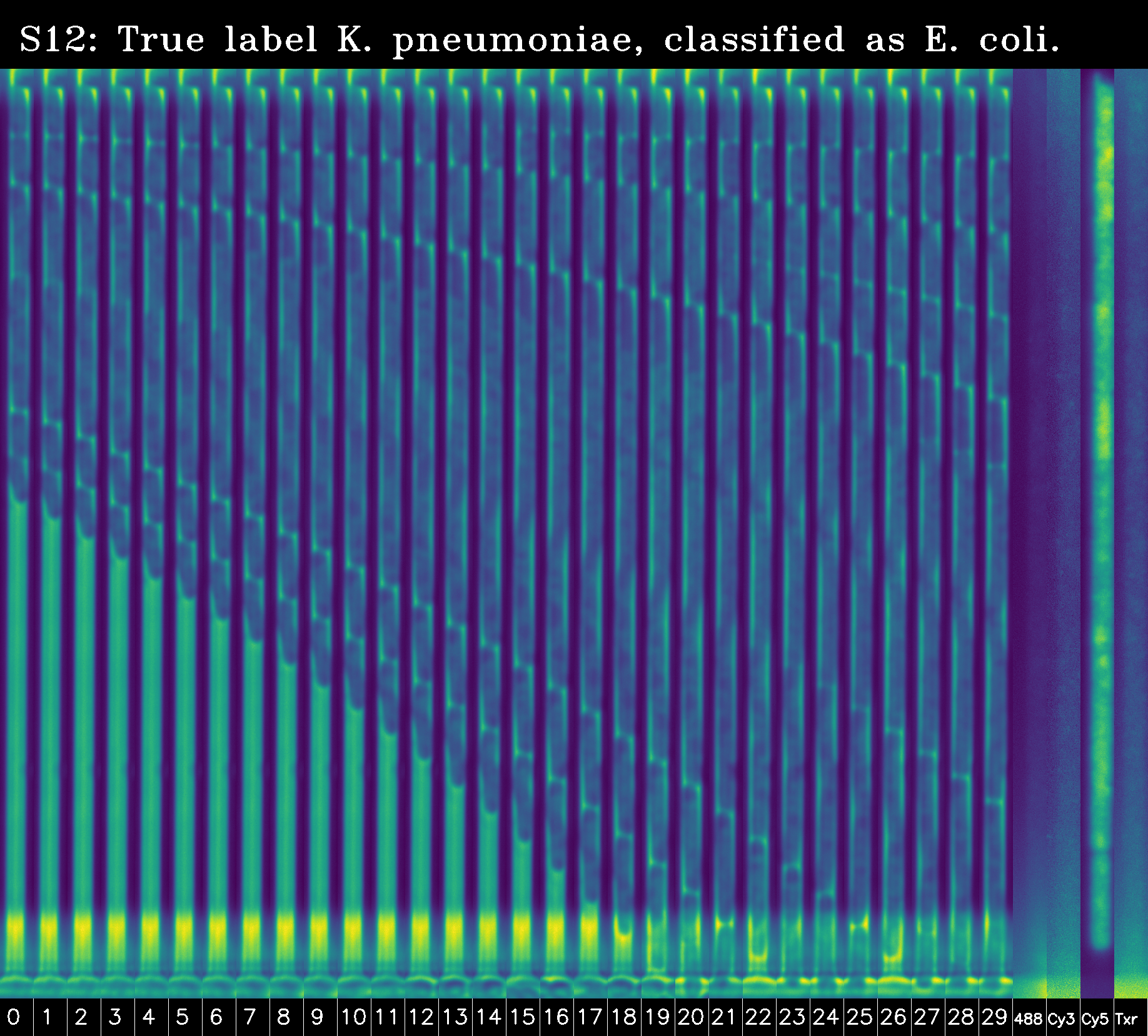

Supplement: S12 Fig — True label K. pneumoniae, classified as E. coli. Both species are rods with similar shapes and are easily confused. (TIF) [file pcbi.1011181.s012.tif]
