# Supplementary material for: Label-free deep learning-based species classification of bacteria imaged by phase-contrast microscopy
Source: PLoS Comput Biol. 2023 Nov 13;19(11):e1011181. doi: 10.1371/journal.pcbi.1011181 (PMC10681317; doi:10.1371/journal.pcbi.1011181)
Supplement: S1 Appendix — A balanced dataset of 250 time-lapses per species was extracted from the untreated samples, trained, and tested on downscaled versions of CIFAIR-10 ResNet and ViT-B/8. (PDF) [file pcbi.1011181.s017.pdf]

S1 Appendix: Fully balanced experiments using non-treated traps

The results showed a consistently low classification accuracy of *Pseudomonas aeruginosa* in the single-frame experiments, especially when using smaller models and subsampled low-resolution images. It was suspected that this could be because this particular species contained only 250 out of 662 untreated time-lapses. In response, we conducted additional experiments, selecting only 250 untreated traps from each species, using a 15% train/test split as before, re-training the downscaled versions of ResNet-8 and ViT-B/8, testing on the first frame. The results showed similar tendencies on this minimal test set. *Pseudomonas aeruginosa* attained the lowest accuracy in 20 and the second lowest in 6 of the models, as shown in Tables 1 and 2.

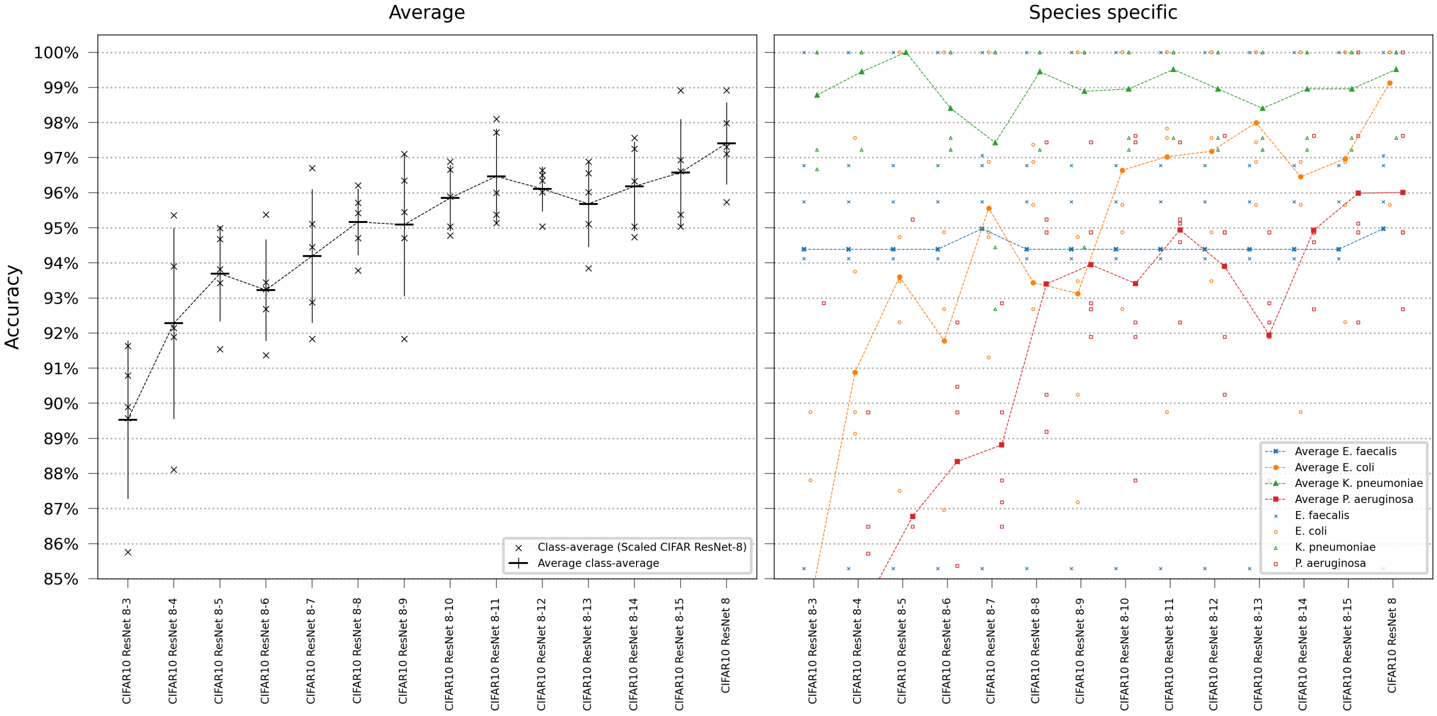

Figure 1: Model comparison performing single-frame classification of the first frame in the time-lapse using downscaled versions of ResNet-8. Error bars represent the standard deviation in class-average accuracy from the five retrainings. Scatter plots depict the class-specific accuracy of all individual classifiers and the average class-specific accuracy over the five retrainings for each model. To reduce overplotting, a minor jitter was introduced along the categorical axis of the species-specific scatter plot. Lines are included not for interpolation or statistical inference purposes but to visually guide readers in tracking mean values on the ordinal scale.

Model name	*E. faecalis*	*E. coli*	*K. pneumoniae*	*P. aeruginosa*
CIFAR10 ResNet 8-3	$94.39 \pm 5.52\%$	$84.31 \pm 5.67\%$	$98.78 \pm 1.69\%$	$80.65 \pm 8.27\%$
CIFAR10 ResNet 8-4	$94.39 \pm 5.52\%$	$90.88 \pm 5.04\%$	$99.44 \pm 1.24\%$	$84.41 \pm 4.49\%$
CIFAR10 ResNet 8-5	$94.39 \pm 5.52\%$	$93.60 \pm 4.50\%$	$100.00 \pm 0.00\%$	$86.78 \pm 4.89\%$
CIFAR10 ResNet 8-6	$94.39 \pm 5.52\%$	$91.78 \pm 6.25\%$	$98.40 \pm 1.47\%$	$88.34 \pm 3.60\%$
CIFAR10 ResNet 8-7	$94.97 \pm 5.64\%$	$95.56 \pm 3.19\%$	$97.43 \pm 3.58\%$	$88.81 \pm 2.57\%$
CIFAR10 ResNet 8-8	$94.39 \pm 5.52\%$	$93.44 \pm 5.26\%$	$99.44 \pm 1.24\%$	$93.40 \pm 3.52\%$
CIFAR10 ResNet 8-9	$94.39 \pm 5.52\%$	$93.13 \pm 4.84\%$	$98.89 \pm 2.48\%$	$93.95 \pm 2.24\%$
CIFAR10 ResNet 8-10	$94.39 \pm 5.52\%$	$96.64 \pm 3.25\%$	$98.96 \pm 1.43\%$	$93.41 \pm 4.15\%$
CIFAR10 ResNet 8-11	$94.39 \pm 5.52\%$	$97.03 \pm 4.23\%$	$99.51 \pm 1.09\%$	$94.94 \pm 1.83\%$
CIFAR10 ResNet 8-12	$94.39 \pm 5.52\%$	$97.18 \pm 2.96\%$	$98.96 \pm 1.43\%$	$93.90 \pm 2.88\%$
CIFAR10 ResNet 8-13	$94.39 \pm 5.52\%$	$97.99 \pm 1.94\%$	$98.40 \pm 1.47\%$	$91.95 \pm 2.58\%$
CIFAR10 ResNet 8-14	$94.39 \pm 5.52\%$	$96.45 \pm 4.21\%$	$98.96 \pm 1.43\%$	$94.93 \pm 1.76\%$
CIFAR10 ResNet 8-15	$94.39 \pm 5.52\%$	$96.97 \pm 3.23\%$	$98.96 \pm 1.43\%$	$95.98 \pm 2.93\%$
CIFAR10 ResNet 8	$94.97 \pm 5.64\%$	$99.13 \pm 1.94\%$	$99.51 \pm 1.09\%$	$96.01 \pm 2.84\%$

Table 1: Model comparison performing single-frame classification testing on the first frame in the time-lapse using downscaled versions of ResNet-8. The table shows species-specific accuracies and standard deviations across the predetermined train/test splits. The lowest species-specific accuracies are highlighted in light blue.

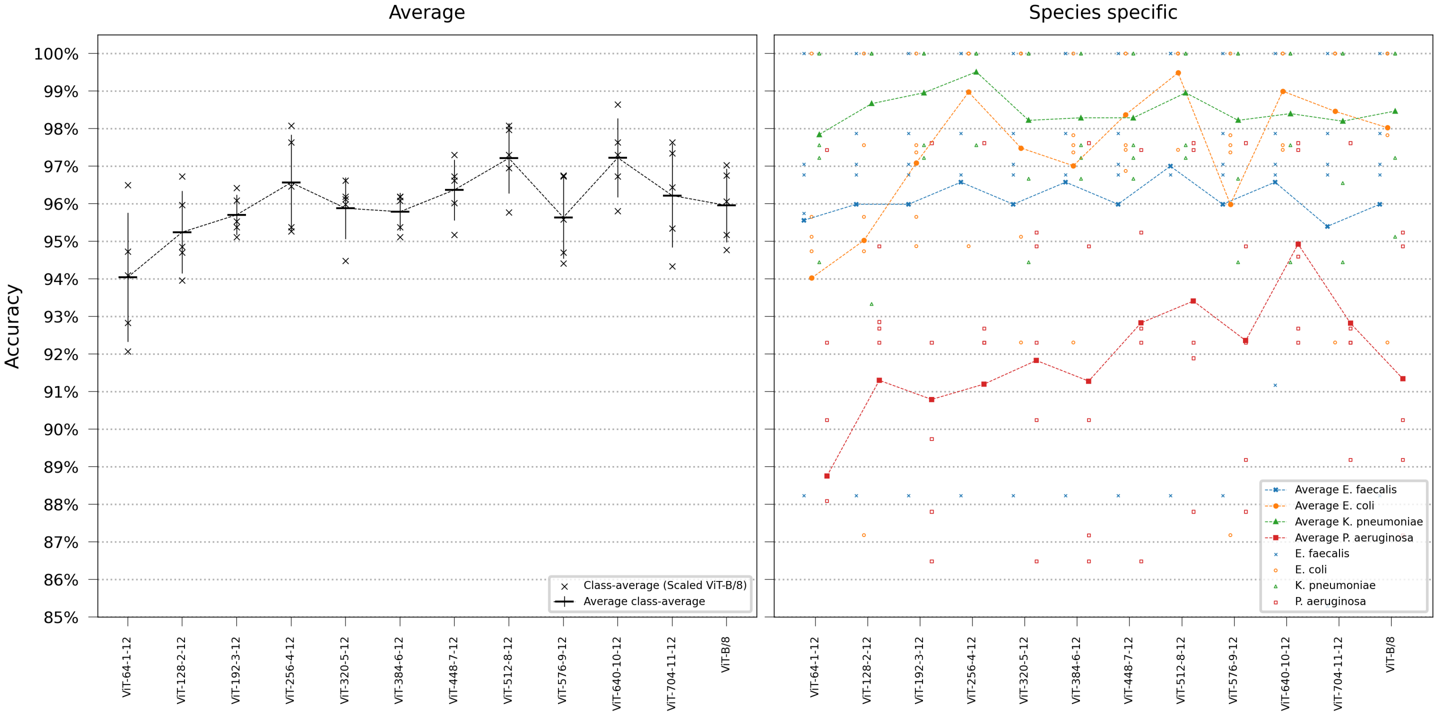

Figure 2: Model comparison performing single-frame classification of the first frame in the time-lapse using downscaled versions of ViT-B/8. Error bars represent the standard deviation in class-average accuracy from the five retrainings. Scatter plots depict the class-specific accuracy of all individual classifiers and the average class-specific accuracy over the five retrainings for each model. To reduce overplotting, a minor jitter was introduced along the categorical axis of the species-specific scatter plot. Lines are included not for interpolation or statistical inference purposes but to visually guide readers in tracking mean values on the ordinal scale.

Model name	*E. faecalis*	*E. coli*	*K. pneumoniae*	*P. aeruginosa*
ViT-64-1-12	$95.56 \pm 4.39\%$	$94.03 \pm 5.67\%$	$97.85 \pm 2.31\%$	$88.75 \pm 8.09\%$
ViT-128-2-12	$95.99 \pm 4.51\%$	$95.03 \pm 4.83\%$	$98.67 \pm 2.98\%$	$91.30 \pm 4.32\%$
ViT-192-3-12	$95.99 \pm 4.51\%$	$97.09 \pm 1.98\%$	$98.96 \pm 1.43\%$	$90.79 \pm 4.40\%$
ViT-256-4-12	$96.58 \pm 4.87\%$	$98.97 \pm 2.29\%$	$99.51 \pm 1.09\%$	$91.20 \pm 6.09\%$
ViT-320-5-12	$95.99 \pm 4.51\%$	$97.49 \pm 3.58\%$	$98.22 \pm 2.56\%$	$91.83 \pm 3.61\%$
ViT-384-6-12	$96.58 \pm 4.87\%$	$97.01 \pm 2.84\%$	$98.29 \pm 1.59\%$	$91.28 \pm 4.85\%$
ViT-448-7-12	$95.99 \pm 4.51\%$	$98.37 \pm 1.51\%$	$98.29 \pm 1.59\%$	$92.83 \pm 4.11\%$
ViT-512-8-12	$97.00 \pm 5.10\%$	$99.49 \pm 1.15\%$	$98.96 \pm 1.43\%$	$93.41 \pm 4.15\%$
ViT-576-9-12	$95.99 \pm 4.51\%$	$95.99 \pm 5.04\%$	$98.22 \pm 2.56\%$	$92.36 \pm 4.02\%$
ViT-640-10-12	$96.58 \pm 3.27\%$	$99.00 \pm 1.37\%$	$98.40 \pm 2.45\%$	$94.93 \pm 2.53\%$
ViT-704-11-12	$95.40 \pm 5.79\%$	$98.46 \pm 3.44\%$	$98.20 \pm 2.58\%$	$92.82 \pm 3.03\%$
ViT-B/8	$95.99 \pm 4.51\%$	$98.03 \pm 3.33\%$	$98.47 \pm 2.22\%$	$91.34 \pm 3.56\%$

Table 2: Model comparison performing single-frame classification testing on the first frame in the time-lapse using downscaled versions of ViT/B-8. The table shows species-specific accuracies and standard deviations across the predetermined train/test splits. The lowest species-specific accuracies are highlighted in light blue.
