# Supplementary material for: Label-free deep learning-based species classification of bacteria imaged by phase-contrast microscopy
Source: PLoS Comput Biol. 2023 Nov 13;19(11):e1011181. doi: 10.1371/journal.pcbi.1011181 (PMC10681317; doi:10.1371/journal.pcbi.1011181)
Supplement: S2 Appendix — Training single-frame ResNet-26 and ViT-16, testing on incrementally later frames. (PDF) [file pcbi.1011181.s018.pdf]

S2 Appendix: Species-specific accuracy plots testing on later frames

The following are species-specific results training pretrained single-frame ResNet-26 and ViT-16, testing on incrementally later frames.

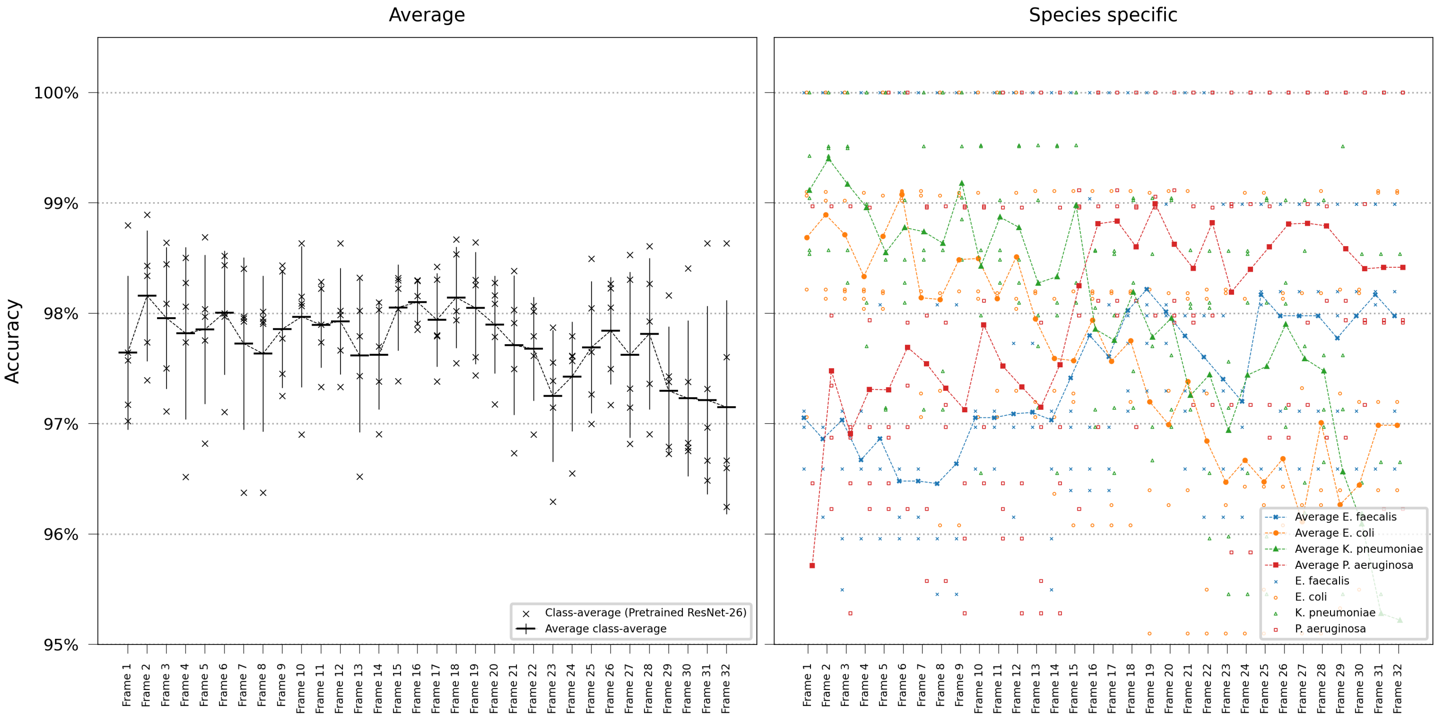

Figure 1: Performing single-frame classification using pretrained ResNet-26, testing on subsequently later frames in the time-lapse. Error bars represent the standard deviation in class-average accuracy from the five retrainings. Scatter plots depict the class-specific accuracy of all individual classifiers and the average class-specific accuracy over the five retrainings for each model. To reduce overplotting, a minor jitter was introduced along the categorical axis of the species-specific scatter plot. Lines are included not for interpolation or statistical inference purposes but to visually guide readers in tracking mean values on the ordinal scale.

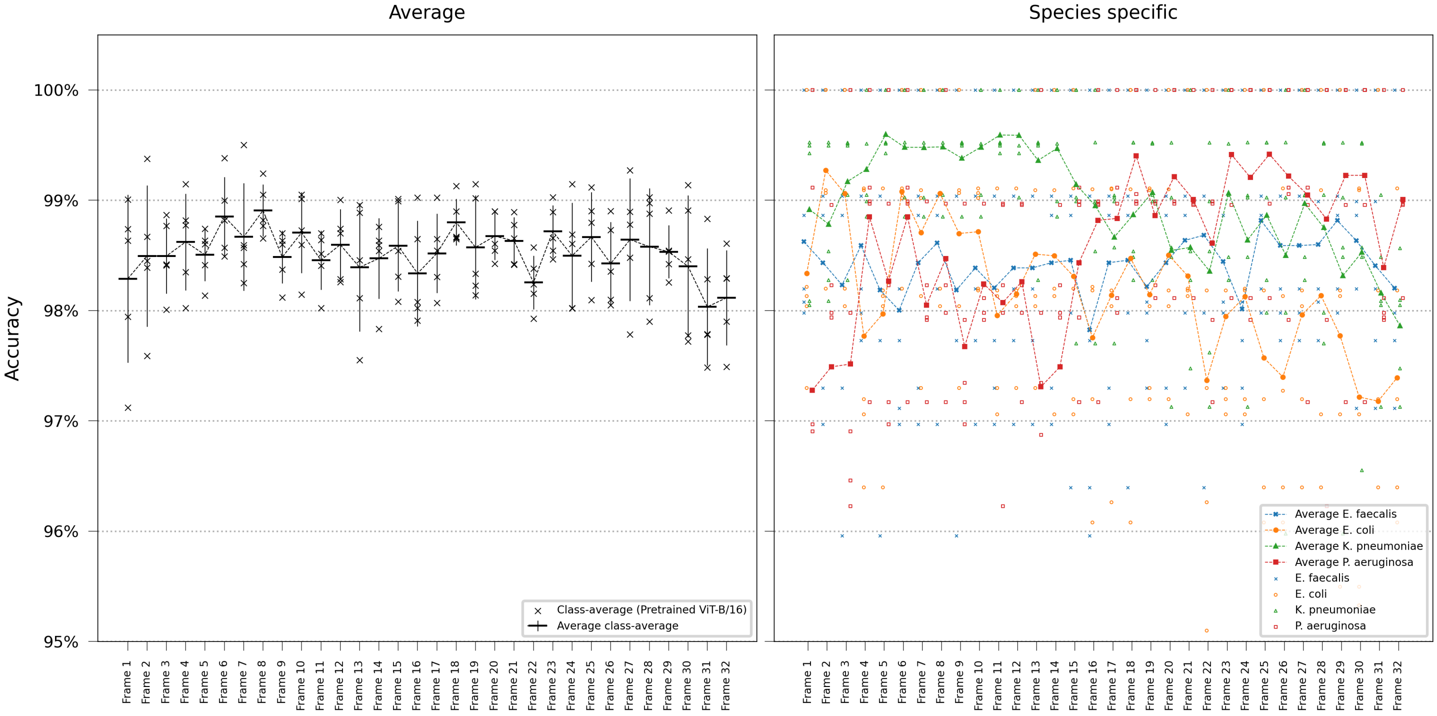

Figure 2: Performing single-frame classification pretrained ViT-8/B, testing on subsequently later frames in the time-lapse. Error bars represent the standard deviation in class-average accuracy from the five retrainings. Scatter plots depict the class-specific accuracy of all individual classifiers and the average class-specific accuracy over the five retrainings for each model. To reduce overplotting, a minor jitter was introduced along the categorical axis of the species-specific scatter plot. Lines are included not for interpolation or statistical inference purposes but to visually guide readers in tracking mean values on the ordinal scale.
