# Supplementary material for: Label-free deep learning-based species classification of bacteria imaged by phase-contrast microscopy
Source: PLoS Comput Biol. 2023 Nov 13;19(11):e1011181. doi: 10.1371/journal.pcbi.1011181 (PMC10681317; doi:10.1371/journal.pcbi.1011181)
Supplement: S3 Appendix — Inference latency measurements (seconds/sample) of the models using our hardware setup. (PDF) [file pcbi.1011181.s019.pdf]

S3 Appendix: Inference latency

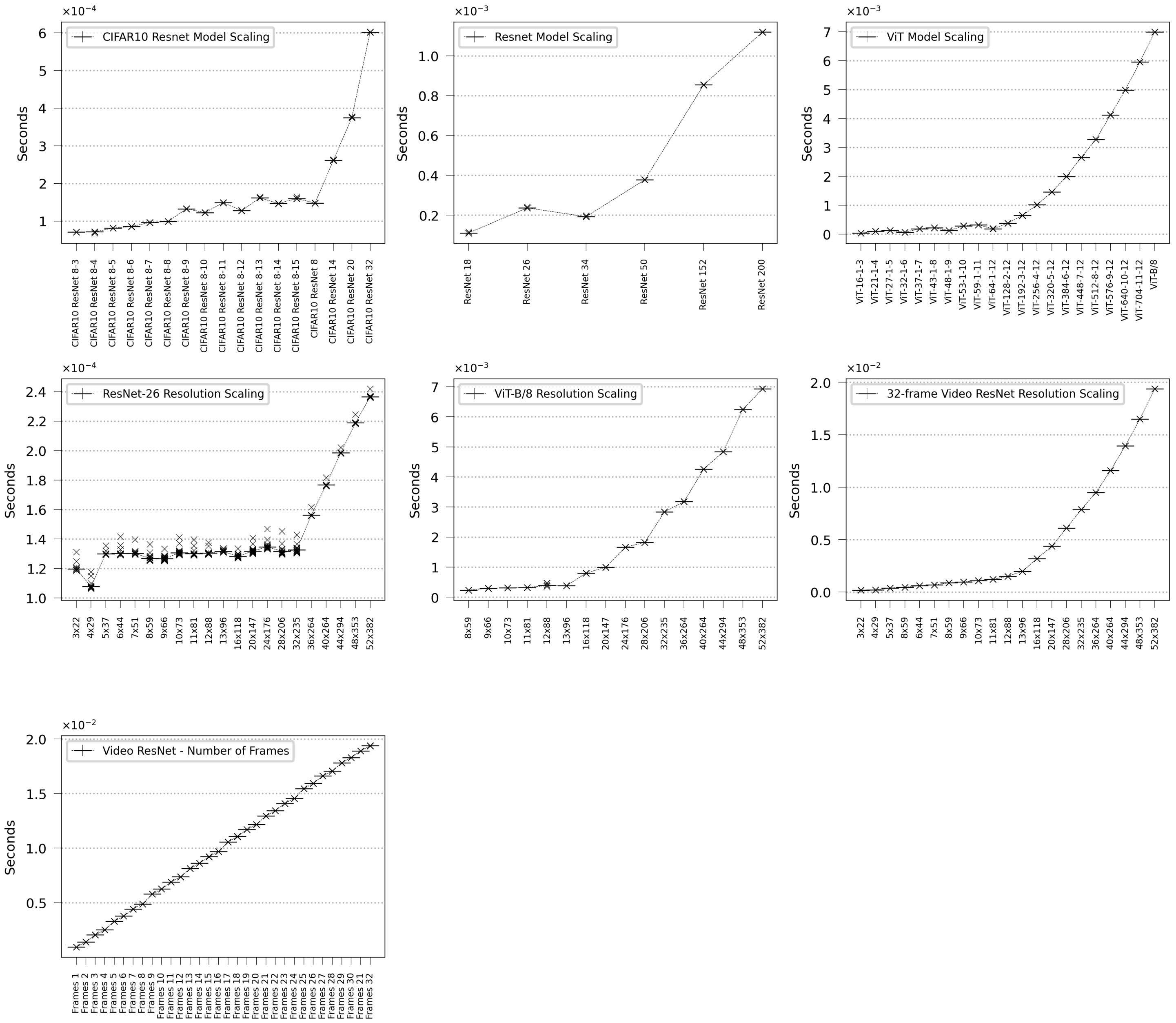

Figure 1: Inference time per sample (using batch-size 32) was measured across all models over 30 batches, following an initial warm-up phase of 30 batches.
